# Supplementary material for: Phototunable self-oscillating system driven by a self-winding fiber actuator
Source: Nat Commun. 2021 May 28;12:3211. doi: 10.1038/s41467-021-23562-6 (PMC8163889; doi:10.1038/s41467-021-23562-6)
Supplement: Supplementary file 1 — Supplementary Information [file 41467_2021_23562_MOESM1_ESM.pdf]

Supplementary Information

for

Phototunable Self-Oscillating System Driven by A Self-Winding Fiber Actuator

by Hu, et al.

Characterization

DSC thermograms were obtained by TA Discovery DSC 250 (Rate of heating: 5 ^{o}C/min, N_2 flow 50 ml/min). Thermal gravity analyses (TGA) were carried out on a Mettler-Toledo TGA/DSC 3+/1600 HT analyzer. Mechanical properties of the LCE fibers were investigated by Instron 5943 universal testing system with the tensile testing rate of 50 mm/min. 2D X-ray diffraction patterns were obtained by using a Bruker D8 Venture diffractometer. The intensity of the concentrated sunlight was measured by a solar power meter (LP-3A, Merry Change). The intensity of the concentrated sunlight used to drive the self-oscillation of PSOS is ~ 3.5 W cm^{-2}. Induced current was monitored by microcurrent sensor (llongwill® LW-E821, resolution 0.01 μA).

Supplementary Figures

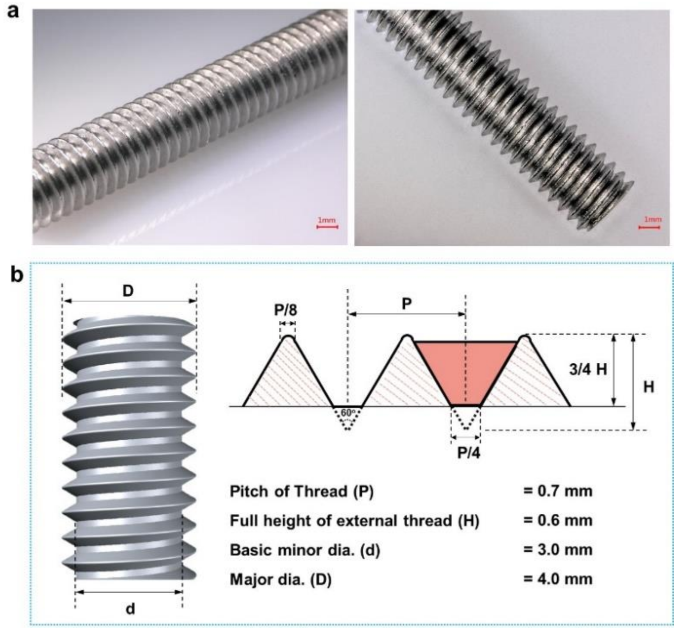

Supplementary Figure 1. **The characteristics of the screw mold**. **a**, Optical photographs showing the screw mold. **b**, Schematics showing the characteristic features of the screw mold.

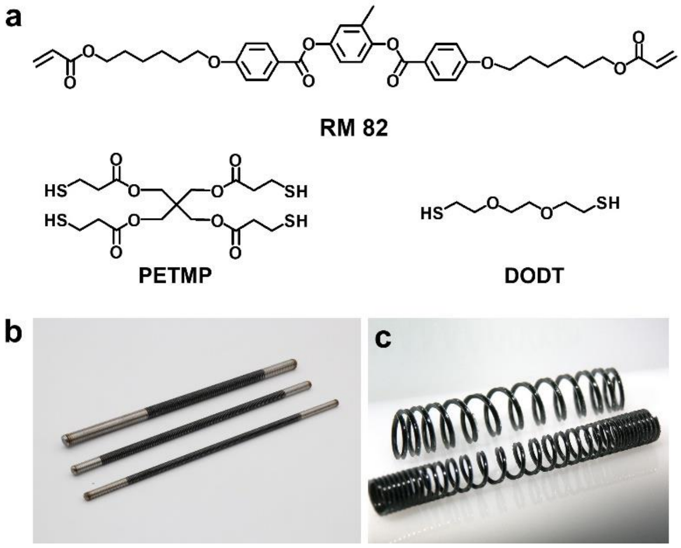

Supplementary Figure 2. Preparation of the precursor for self-winding fiber actuators. a, Chemical structures of used monomers. **b**, photograph showing the soft springs with screw mold. **c**, photograph showing the prepared soft spring after removed the screw mold.

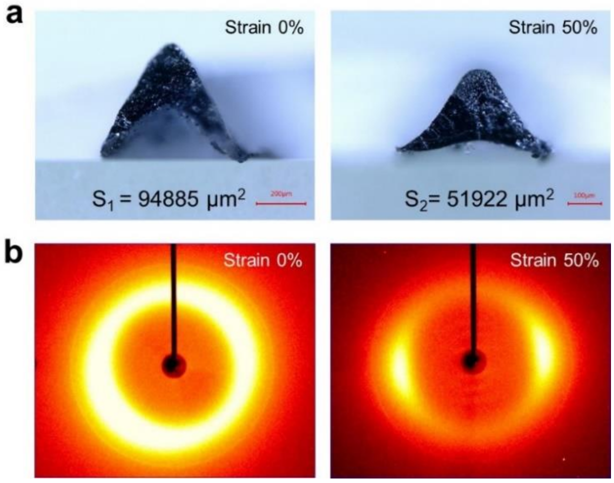

Supplementary Figure 3. LC Alignment through stretching treatment. a, photographs showing the cross section of the soft spring fiber before (left) and after (right) the stretching treatment. S_1 and S_2 indicate the cross-sectional area before and after the stretching treatment. **b**, 2D-WXRD patterns of the soft spring fiber before (left) and after (right) the stretching treatment.

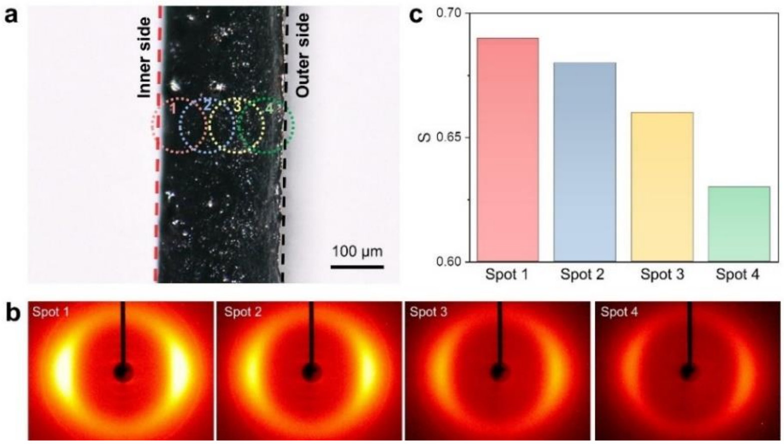

Supplementary Figure 4. Gradient in order parameter over the fibre cross-section. a. photograph showing four areas enclosed by the four colored circles at equal distance interval from the inner side to the outer side of the fiber, which were irradiated and tested in the 2D XRD measurement. **b**, 2D XRD patterns for the four areas indicated by the four colored circles on the fiber. **c**, Evolution of the degree of order over the fibre cross-section. 2D XRD pattern of the inner side of the fiber clearly shows symmetrical diffraction spots, which indicates a high degree of order. As the X-ray spot moves from the inner side to the outer side direction, the diffraction spots become diffuse, suggesting that the degree of order in the fiber gradually decreases from the inner side to the outer side, and a gradient of order parameter exists over the fibre cross-section.

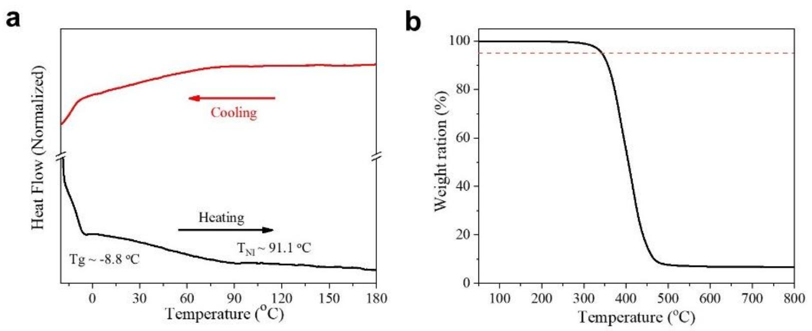

Supplementary Figure 5. **Thermal stability**. **a**, DSC curves of the SWFA. b, TGA curves of the SWFA. Under N_2 atmosphere, the onset temperature of 5 % weight loss was approximately 342 °C for the SWFA.

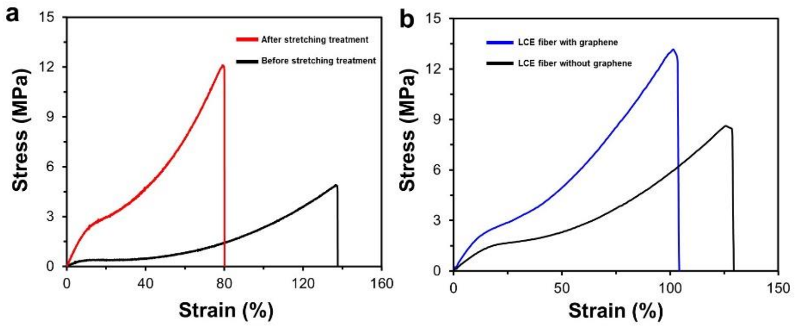

Supplementary Figure 6. Mechanical properties. a, Strain/stress curve of the soft spring precursor before and after the stretching treatment. **b,** Strain/stress curves of self-winding fiber actuator with and without graphene.

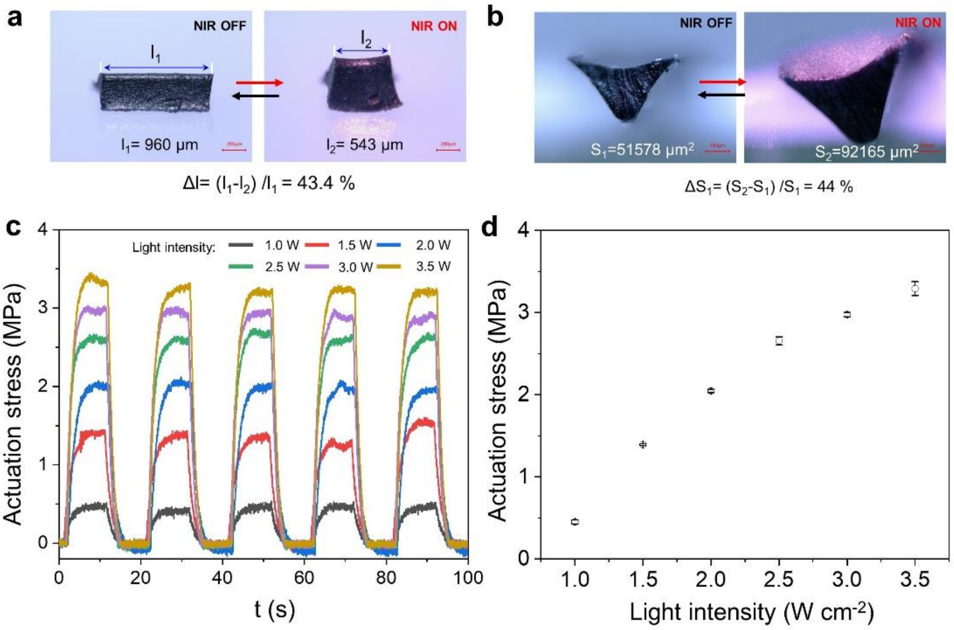

Supplementary Figure 7. Photoactuation of self-wind fiber actuator. a, Lateral photographs showing the light-induced reversible deformation of a piece of SWFA. I_2 and I_1 is the length of the piece of SWFA with and without the NIR irradiation. **b**, Photographs showing the light-induced reversible deformation of the cross section of SWFA. S_2 and S_1 indicate the cross-sectional area of SWFA with and without the NIR irradiation. The intensity of the NIR light is ~ 3 W cm^{-2}. **c**, Actuation stress of SWFA under repeated on (10 s) and off (10 s) cycles with different light intensities. **d**, Actuation stress of SWFA upon varying light intensities. Error bars represent one standard deviation of the data away from the mean.

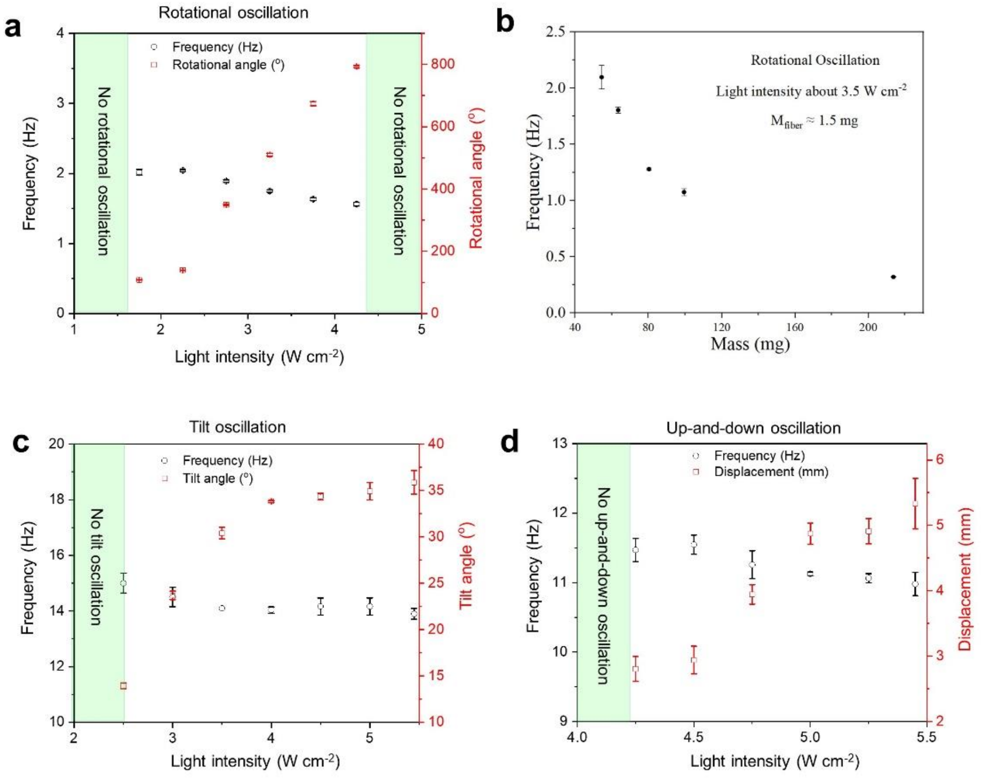

Supplementary Figure 8. Analysis of self-oscillations. a, Evolution of the frequency and the amplitude (rotational angle) of rotational oscillation along with increase of light intensity**. b,** Frequency changes of rotational oscillation as a function of the mass of the hanging load. **c**. Frequency and amplitude (tilt angle) of tilt oscillation as a function of light intensity. The size of light spot is 1.0 mm × 10 mm (Width × Length). **d,** Frequency and amplitude (displacement) of up-and-down oscillation as a function of light intensity. The size of light spot used in the tests is 1.5 mm × 10 mm (Width × Length). Error bars represent one standard deviation of the data away from the mean.

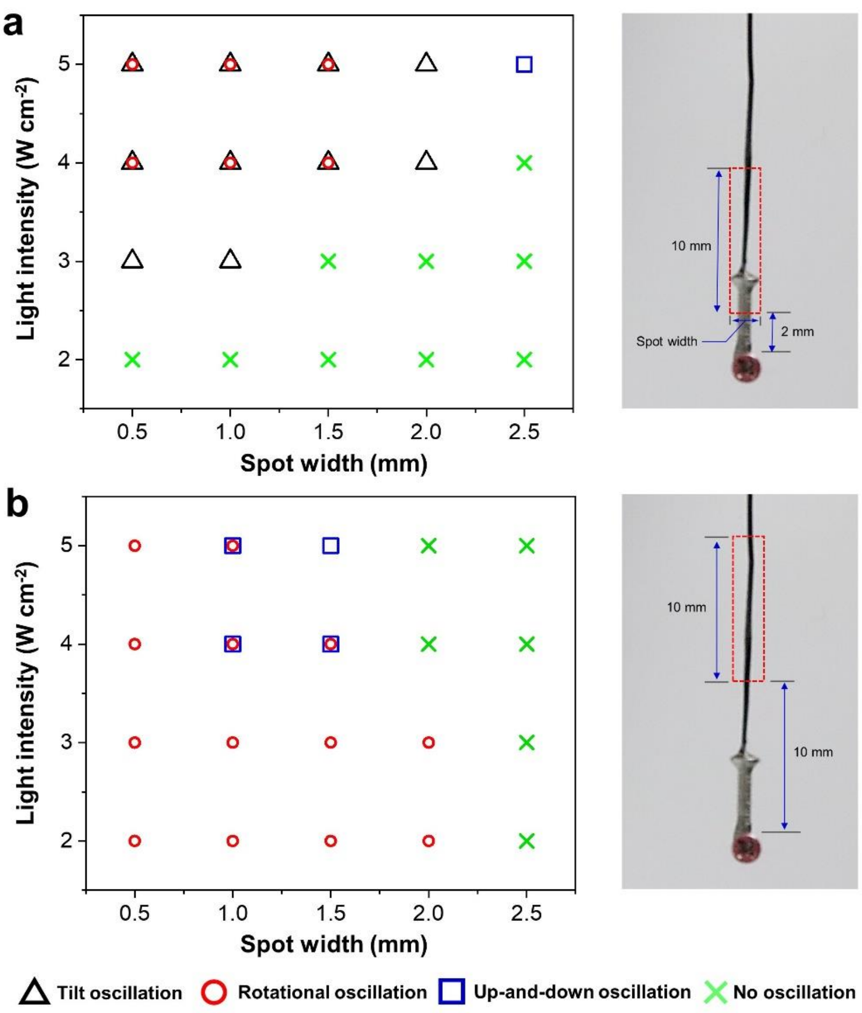

Supplementary Figure 9. Oscillation modes determined by light intensity and width of light spot. a, b, Phase diagram showing diverse oscillations with the variation of light intensity and spot width. The right photographs show the corresponding location and size of the light spot on the SWFA by red dashed box in the tests. The location and length of the light spot on the SWFA are maintained unchanged during the tests. To generate tilt oscillation, the connection between the SWFA and the hanging object must be irradiated. The length of light spot is 10 mm.

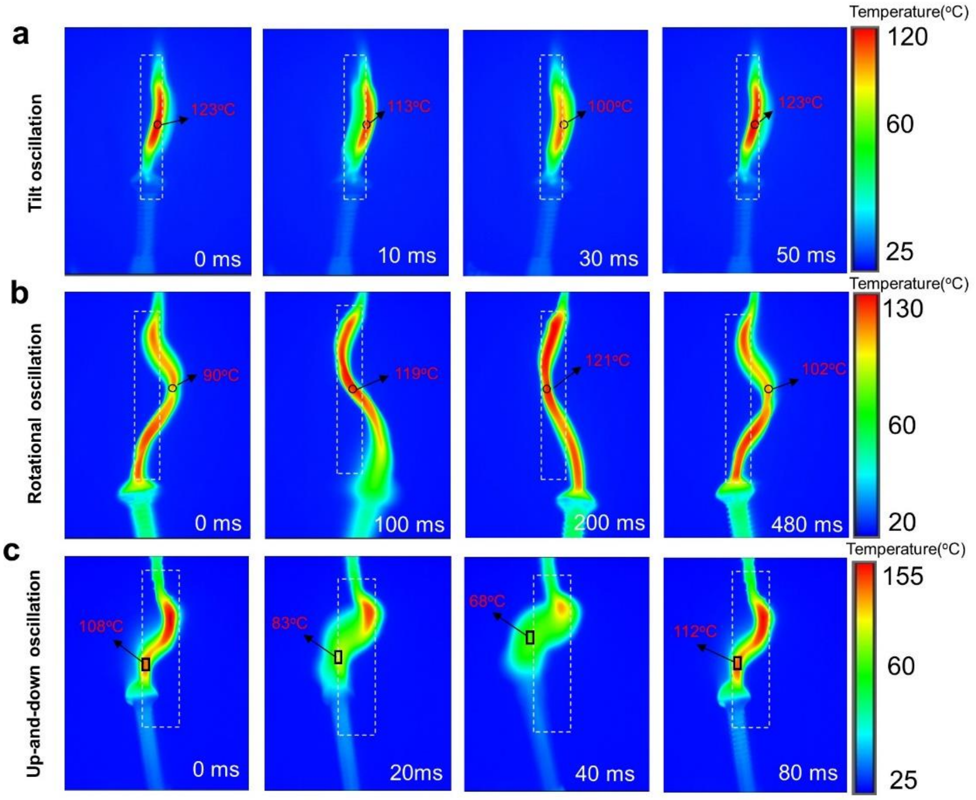

Supplementary Figure 10. Thermal imaging of SWFA in self-oscillations. Snapshots showing the temperature change and distribution on the SWFA during tilt oscillation (**a**), rotational oscillation (**b**), and up-and-down oscillation (**c**), respectively. The temperature of a marked point indicated by a black circle on the SWFA were tracked during tilt oscillation, rotational oscillation, respectively. The maximum temperature of an area enclosed by a black square was tracked in up-and-down oscillation. The white dashed boxes represent the irradiated area by NIR light. The intensity of NIR light is ~ 3.5 W cm^{-2} for tilt oscillation and rotational oscillation, and ~ 4 W cm^{-2} for up-and-down oscillation.

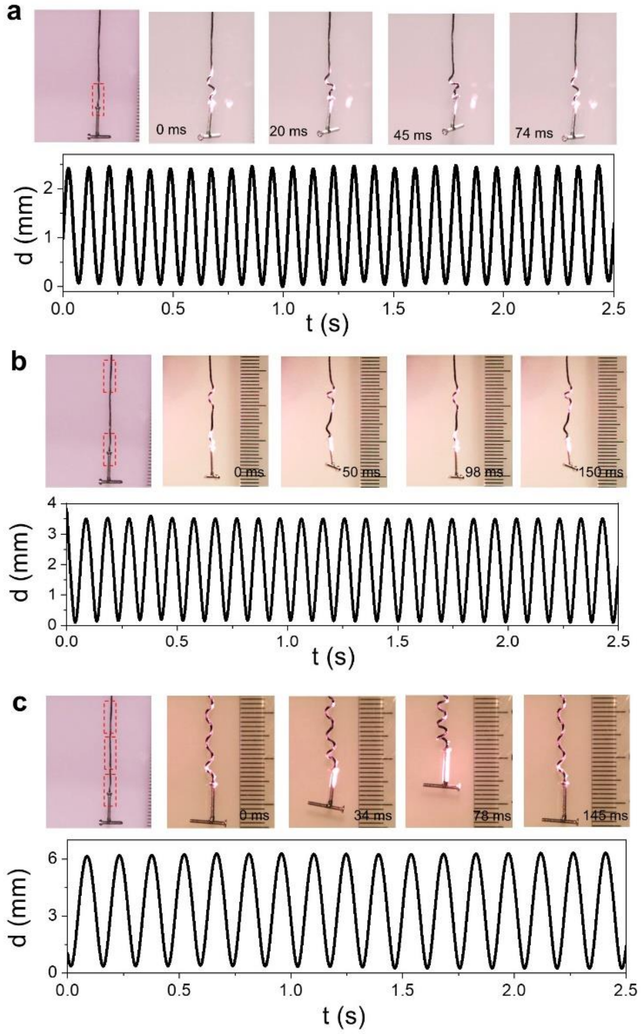

Supplementary Figure 11. Resonance-enhanced Up-and-down oscillation. a, Photographs showing up-and-down oscillation and corresponding dynamics (Left half of the video of Supplementary Movie 8). The amplitude of the oscillation is ± 1.2 mm. **b**, Photographs showing resonance-enhanced up-and-down oscillation and corresponding oscillation dynamics. Two light beams were used to illuminate two different parts of a SWFA. The one part located at the higher position was irradiated by a wide enough spot that can make the irradiated part deform into a stable coiled spring but without oscillation, while the other part near the hanging object was illuminated to induce a coiled spring with time-varying morphologies and generate up-and-down oscillation. The coupling of the two light-induced coiled springs connected in series yields the resonance that amplifies the amplitude of the up-and-down oscillation. The amplitude of the oscillation is ± 1.8 mm. **c**, Photographs showing resonance-enhanced up-and-down oscillation and corresponding oscillation dynamics. Three light beams were used to illuminate three different parts of a SWFA. The two adjacent parts located at the higher positions were irradiated by two wide enough light spots that can make the parts deform into stable coiled springs but without oscillation, while the one part near the hanging object was irradiated to form a coiled spring with time-varying morphologies and generate up-and-down oscillation. The coupling of the three light-induced coiled springs connected in series yields the resonance that significantly amplifies the amplitude of the up-and-down oscillation (Right half of the video of Supplementary Movie 8). The amplitude of the oscillation is ± 3.1 mm. The red dashed boxes in the images indicate the location of the NIR light spots. The mass of the SWFA and the load is ~ 3 mg and ~ 62 mg, respectively. The length of the straight SWFA is ~ 4 cm. The intensity of the NIR light is ~ 3.5 W cm^{-2}.

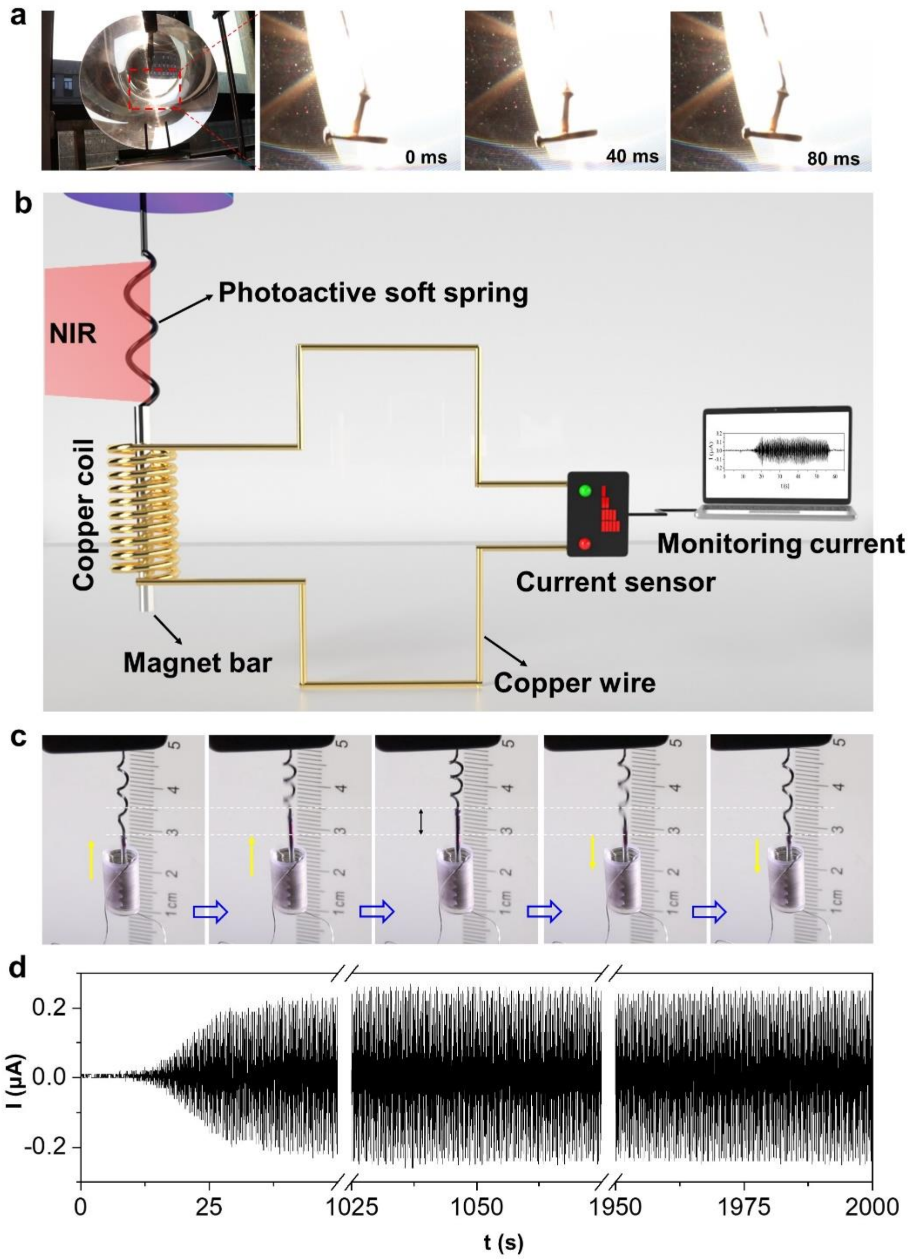

Supplementary Figure 12. Solar capture and conversion. **a**, Autonomous and self-sustainable motion upon the irradiation of normal sunlight. The first image shows the experimental setup located near a window of our lab. The rest three images show the sun-light-driven oscillation. The sunlight focused by a Fresnel lens placed between the window and the actuator fuels the oscillation. **b**, Schematics showing the experimental setup for photon-mechano-electron conversion based on the Faraday's law of electromagnetic induction. **c**, Snapshots show that a SWFA is motorizing the up-and-down motion of a magnetic bar in the cavity of metal coils. **d**, Induction current generated by the experimental setup shown in **b**. The intensity of the NIR light is ~ 3.5 W cm^{-2}.

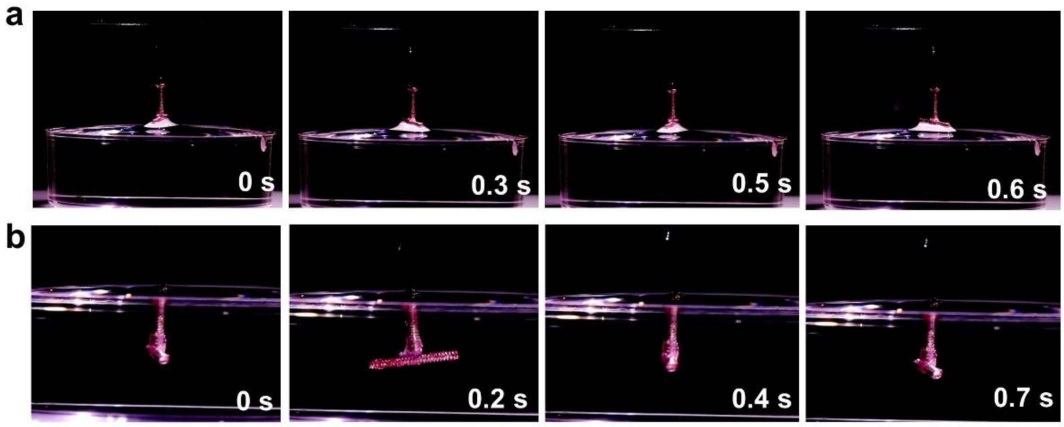

Supplementary Figure 13. Self-oscillation in damping media. a, Photographs showing light-driven rotational oscillation just above water surface. A meniscus is formed between water surface and the hanging object. **b**, Photographs exhibiting light-driven rotational oscillation in water. The mass of the fiber and the load are 1.5 mg and 73 mg, respectively. The intensity of 808-nm light is ~ 3.5 W cm^{-2}.

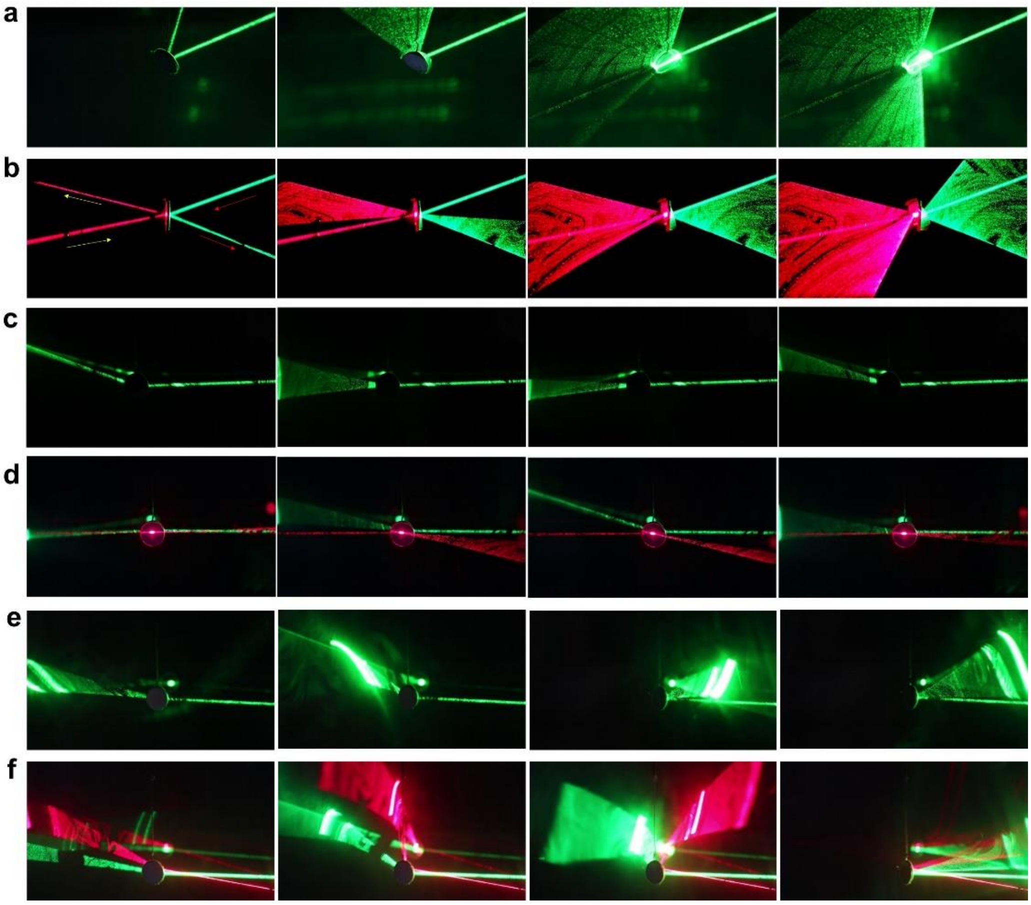

Supplementary Figure 14. Beam steering. a, b, Steering one laser beam and two laser beams by rotational oscillation, respectively. **c, d,** Fast scanning of one laser beam and two laser beams by tilt oscillation, respectively. **e, f,** 2D scanning of single laser beam and two laser beams, respectively.

Supplementary Note

Laser beams

The light used to actuate the fiber actuator is 808-nm NIR laser, which is produced by two types of semiconductor laser diodes. One uses AlGaInP semiconductor (laser pointer, HW808AD1200-22FGD, Shenzhen Infrared Laser Technology Co., Ltd), the other employs AlGaAs semiconductor (PSU-H-LED, MDL-H-808-5W, Changchun New Industries Optoelectronics Technology Co., Ltd). We measured the profiles of these two lasers by a camera-based beam profiler (BeamOn-U3-VIS-NIR, Duma Optronics Ltd). Laser intensities were measured by a high-precision laser power meter (TP100, Changchun New Industries Optoelectronics Technology Co., Ltd). We also carried out laser spectral analysis by a high-resolution spectrometer (Aurora4000, Changchun New Industries Optoelectronics Technology Co., Ltd.). The results of the measurements are shown below:

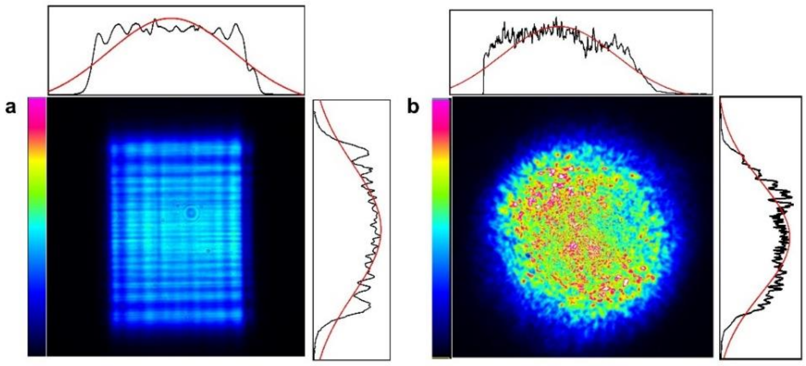

Supplementary Figure 15. Beam profiles of two 808-nm lasers generated by the semiconductor laser diodes of AlGaInP (a) and AlGaAs (b), respectively. The curves near the photos show the intensity distributions of the lasers.

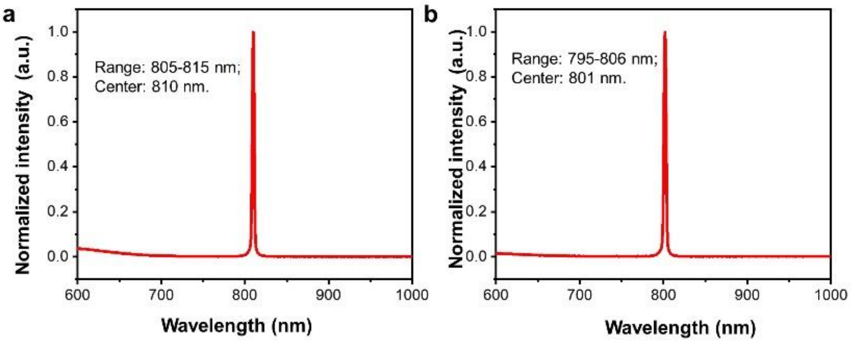

Supplementary Figure 16. Laser spectral analysis of two 808-nm laser beams generated by the semiconductor laser diodes of the AlGaInP (a) and AlGaAs (b), respectively.

Three lights used to demonstrate the steering function exhibited in Fig.5 in the manuscript are 450-nm laser (blue), 532-nm laser (green), and 650-nm laser (red), which are gained by commercial laser pointers purchased from Beijing Huisite Technology Co., Ltd (Green and red laser pointers) and Shenzhen Xinfei Optoelectronics Technology Co., Ltd (Blue laser pointer). The beam profiles of the three lasers, as well as their spectral analysis, are shown below:

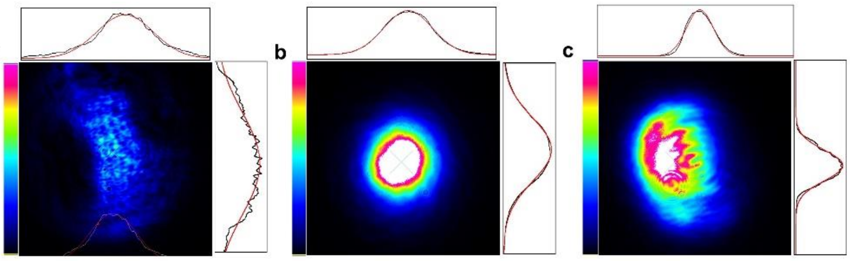

Supplementary Figure 17. Beam profiles of 450-nm laser (a), 532-nm laser (b), and 650-nm laser (c). The curves near the photos show the intensity distributions of the lasers.

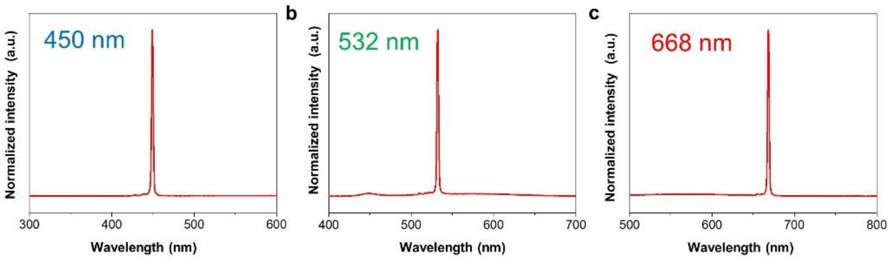

Supplementary Figure 18. Laser spectral analysis of 450-nm laser (a), 532-nm laser (b), and 650-nm laser (c).

Optical absorption coefficient

808-nm NIR light was used to actuate the LCE fiber. The absorption spectra of the LCE film with (2 wt%) and without graphene are shown in Supplementary Fig. 19. From these spectra, we can obtain the absorbance (***A***) of the LCE film with and without graphene at the wavelength of 808 nm.

One of the important parameters determining the optical properties of a material is the optical absorption coefficient (α), which demonstrates the ability of a material to absorb the light of a given wavelength. The change of α according to ***A*** is given by [1],

$$\alpha = 2.303\ \frac{A}{d} \tag{1}$$

Where *A* is the absorbance, and *d* is the thickness of the sample. The wavelength dependence of the α value obtained by using Eq. 1 is given in Supplementary Fig. 19c**.** The optical absorption coefficient (α) of the LCE film with (2 wt%) and without graphene at the wavelength of 808 nm is exhibited in Supplementary Table 1.

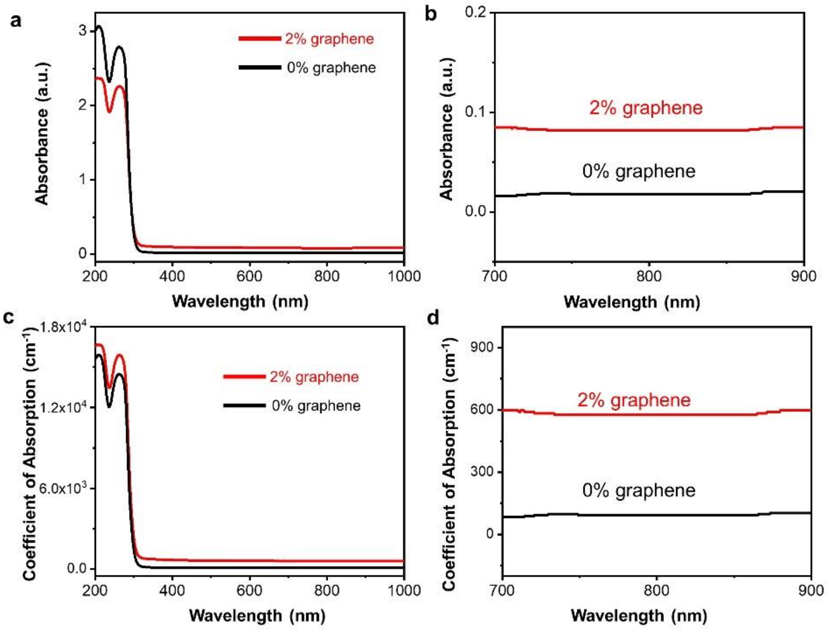

Supplementary Figure 19. **Optical absorption spectra. a, b,** Absorption spectra of LCE film with (2 wt%) and without graphene. **c, d,** Evolution of absorption coefficient of LCE film with (2 wt%) and without graphene along with increase of wavelength.

Supplementary Table 1. Absorption coefficient of the LCE film with and without graphene

Graphene mass fraction	Film thickness [a] (μm)	Coefficient of Absorption (cm^{-1})
0%	4.44 ±0.11	93.4
2%	3.28 ±0.15	576.5

[a] Average value of film thickness of three different positions on the film.

Supplementary Reference

1. Mergen, Ö. B., Arda, E., Kara, S. & Pekcan, Ö. Effects of GNP addition on optical properties and band gap energies of PMMA films. *Polym. Composite.* **40**, 1862-1869 (2019).
