# Supplementary material for: Phototunable self-oscillating system driven by a self-winding fiber actuator
Source: Nat Commun. 2021 May 28;12:3211. doi: 10.1038/s41467-021-23562-6 (PMC8163889; doi:10.1038/s41467-021-23562-6)
Supplement: Supplementary file 3 — Description of Additional Supplementary Files [file 41467_2021_23562_MOESM3_ESM.pdf]

Description of Additional Supplementary Files

File Name: Supplementary Movie 1
Description: **Light-driven shape transformation with high degrees of freedom.** Light-driven shape transformation of SWFA from a straight structure into a helically coiled structure exhibits diverse deformations with multiple DOFs, such as twisting, bending, coiling, and winding. The intensity of 808-nm light is ~ 4 W cm-2 , the length and cross-sectional area of the fiber actuator is 2 cm and 0.046 mm2 , respectively. The mass of the fiber and the load is 1.2 mg and 35 mg, respectively.

File Name: Supplementary Movie 2
Description: **Tilt oscillation.** To gain tilt oscillation, the connection part between the hanging object and the SWFA must be irradiated while the light intensity is required to above a threshold. The intensity of 808-nm light is ~ 3 W cm-2 , the length and crosssectional area of the fiber actuator is 2.5 cm and 0.046 mm2 , respectively. The mass of the fiber and the load is 1.5 mg and 62 mg, respectively.

File Name: Supplementary Movie 3
Description: **Rotational oscillation.** Part1. The intensity of 808-nm light is ~ 2 W cm-2 ; Part2. The intensity of 808-nm light is ~ 3.5 W cm-2 . The length and cross-sectional area of the fiber actuator is 2.5 cm and 0.046 mm2 , respectively. The mass of the fiber and the load is 1.5 mg and 62 mg, respectively

File Name: Supplementary Movie 4
Description: **Up-and-down oscillation.** The intensity of 808-nm light is ~ 4.5 W cm-2 , the length and cross-sectional area of the fiber actuator is 2.5 cm and 0.046 mm2 , respectively. The mass of the fiber and the load is 1.5 mg and 70 mg, respectively.

File Name: Supplementary Movie 5
Description: **Phototunable self-oscillation.** Part 1. Up-and-down oscillation Tilt oscillation Rotational oscillation Part 2. Up-and-down oscillation Rotational oscillation Tilt oscillation Part 3. Tilt oscillation Up-and-down oscillation Rotational oscillation Part 4. Rotational oscillation Tilt oscillation Up-and-down oscillation Phototunable self-oscillating system (PSOS) can flexibly switch among the three basic oscillating modes by changing the location of the NIR spot and the light intensity. The intensity of 808-nm light is ~ 3.5 W cm-2 for tilt oscillation and rotational oscillation and ~ 5 W cm-2 for up-and-down oscillation. The length and cross-sectional area of the fiber is 2.5 cm and 0.046 mm2 , respectively. The mass of the fiber and the load is 1.5 mg and 62 mg, respectively.

File Name: Supplementary Movie 6
Description: **Moving in and out of the light field during self-oscillation.** Part 1. Moving in and out of the light field during tilt oscillation Part 2. Moving in and out of the light field during rotational oscillation Part 3. Moving in and out of the light field during up-and-down oscillation The intensity of 808-nm light is ~ 3.5 W cm-2 for tilt oscillation and rotational oscillation and ~ 5 W cm-2 for upand-down oscillation. The red dashed boxes indicate the irradiated area. The length and cross-sectional area of the fiber actuator is 2.5 cm and 0.046 mm2 , respectively. The mass of the fiber and the load is 1.5 mg and 62 mg, respectively.

File Name: Supplementary Movie 7
Description: **Temperature change in self-oscillation** Part 1. Temperature distribution and change in tilt oscillation Part 1. Temperature distribution and change in rotational oscillation Part 1. Temperature distribution and change in up-and-down oscillation The intensity of 808-nm light is ~ 3.5 W cm-2 for tilt oscillation and rotational oscillation and ~ 4 W cm-2 for upand-down oscillation. The red dashed boxes indicate the irradiated area. The length and cross-sectional area of the fiber actuator is 2.5 cm and 0.046 mm2 , respectively. The mass of the fiber and the load is 1.5 mg and 62 mg, respectively

File Name: Supplementary Movie 8
Description**: Autonomous and sustainable self-oscillation fueled by normal sunlight irradiation.** The sunlight focused by a Fresnel lens fuels the self-oscillation. The length and cross-sectional area of the fiber actuator is 2.5 cm and 0.046 mm2 , respectively. The mass of the fiber and the load is 1.5 mg and 62 mg, respectively.

File Name: Supplementary Movie 9
Description: **Light-driven self-oscillations in damping media. Part** 1. Light-driven rotational oscillation in damping media Part 2. Light-driven tilt oscillation in damping media The intensity of 808-nm light is ~ 3.5 W cm-2 , the length and cross-sectional area of the fiber is 2.5 cm and 0.046 mm2 , respectively. The mass of the fiber and the load is 1.5 mg and 62 mg, respectively

File Name: Supplementary Movie 10
Description: **Amplitude amplification of up-and-down oscillation by resonance.** The intensity of 808-nm light is ~ 4 W cm-2 , the length and cross-sectional area of the fiber is 5 cm and 0.046 mm2 , respectively. The mass of the fiber and the load is 3 mg and 62 mg, respectively.

File Name: Supplementary Movie 11
Description: **Complex oscillation that combines rotational oscillation and tilt oscillation.** The intensity of 808-nm light is ~ 3 W cm-2 , the length and cross-sectional area of the fiber is 2.5 cm and 0.046 mm2 , respectively. The mass of the fiber and the load is 1.5 mg and 62 mg, respectively

File Name: Supplementary Movie 12
Description: **Complex oscillation that combines rotational oscillation and up-and-down oscillation.** The intensity of 808-nm light is ~ 4.5 W cm-2 , the length and cross-sectional area of the fiber is 2.5 cm and 0.046 mm2 , respectively. The mass of the fiber and the load is 1.5 mg and 62 mg, respectively.

File Name: Supplementary Movie 13
Description: **Beam Steering driven by rotational oscillation.** The intensity of 808-nm light is ~ 3.5 W cm-2 , the length and cross-sectional area of the fiber is 2.5 cm and 0.046 mm2 , respectively. The mass of the fiber is 1.5 mg. The diameter of the mirror is 1cm.

File Name: Supplementary Movie 14
Description: **Fast laser scanning driven by tilt oscillation. The** intensity of 808-nm light is ~ 3.5 W cm-2 , the length and cross-sectional area of the fiber is 2.5 cm and 0.046 mm2 , respectively. The mass of the fiber is 1.5 mg. The diameter of the mirror is 1 cm

File Name: Supplementary Movie 15

Description: **Two-dimensional scanning driven by complex self-oscillation.** The intensity of 808-nm light is ~ 3.5 W cm-2 , the length and cross-sectional area of the fiber is 2.5 cm and 0.046 mm2 , respectively. The mass of the fiber is 1.5 mg. The diameter of the mirror is 1 cm.
